# Sex-Dependent Correlations between the Personality Dimension of Harm Avoidance and the Resting-State Functional Connectivity of Amygdala Subregions

**DOI:** 10.1371/journal.pone.0035925

**Published:** 2012-04-27

**Authors:** Ying Li, Wen Qin, Tianzi Jiang, Yunting Zhang, Chunshui Yu

**Affiliations:** 1 Department of Radiology, Tianjin Medical University General Hospital, Tianjin, China; 2 LIAMA Center for Computational Medicine, National Laboratory of Pattern Recognition, Institute of Automation, Chinese Academy of Sciences, Beijing, China; Hangzhou Normal University, China

## Abstract

Harm avoidance (HA) is a personality dimension involving the tendency to respond intensely to signals of aversive stimuli. Many previous neuroimaging studies have associated HA scores with the structural and functional organization of the amygdala, but none of these studies have evaluated the correlation between HA score and amygdala resting-state functional connectivity (rsFC). Moreover, the amygdala is not a homogeneous structure, and it has been divided into several structurally and functionally distinct subregions. Investigating the associations between HA score and properties of subregions of the amygdala could greatly improve our understanding of HA. In the present study, using a large sample of 291 healthy young adults, we aimed to uncover correlations between HA scores and the rsFCs of each amygdala subregion and to uncover possible sex-based differences in these correlations. We found that subregions of the amygdala showed different rsFC patterns, which contributed differently to individual HA scores. More specifically, HA scores were correlated with rsFCs between the laterobasal amygdala subregion and temporal and occipital cortices related to emotional information input, between the centromedial subregion and the frontal cortices associated with emotional output control, and between the superficial subregion and the frontal and temporal areas involved in both functions. Moreover, significant gender-based differences were uncovered in these correlations. Our findings provide a more detailed model of association between HA scores and amygdala rsFC, extend our understanding of the connectivity of subregions of the amygdala, and confirm sex-based differences in HA associations.

## Introduction

Harm avoidance (HA) is a personality dimension derived from the Tridimensional Personality Questionnaire (TPQ) and the Temperament and Character Inventory (TCI) and is considered to be a heritable tendency to respond intensely to signals of aversive stimuli, which causes the inhibition of behaviors leading to punishment, novelty or frustration [1], [2]. Individuals with high HA scores are characterized as worrisome, cautious, apprehensive, pessimistic, shy and easily fatigued [1], [2], and they often show high risks for depression- [3]–[5] and anxiety-related disorders [6]–[9]. Many researchers have investigated the potential neural substrates of HA and have proposed the amygdala as a likely candidate.

The amygdala has been implicated in a wide range of functions, including social behavior, emotional and reward learning, and in particular, aversive emotional processing [10]–[12]. An association between the architecture of the amygdala and individual HA scores has long been appreciated. Structurally, HA score is positively correlated with left-amygdala volume in healthy females [13]; however, neuroticism scores are negatively correlated with right-amygdala gray-matter concentration [14]. It should be noted that the HA and neuroticism are not entirely congruent because the former reflects a tendency to respond intensely to aversive stimuli [1], [2] while the latter reflects a tendency to experience negative emotions [15], although both of them are correlated with each other [16] and are associated with anxiety- and depression-related disorders [3]–[9], [17]. Functionally, HA score is negatively correlated with left-amygdala activation in healthy females viewing negatively valenced baby faces [18], but individuals having high HA scores demonstrate greater amygdala activation when maintaining a specific attention set [19]. The amygdala is also an important node in the corticolimbic circuit, and functional coupling within this circuit is associated with individual HA scores [20]. However, no published studies have yet evaluated the correlation between HA scores and amygdala resting-state functional connectivity (rsFC). Additionally, the amygdala might play an important role in mediating the association between high HA scores and depression/anxiety disorders, as alterations in amygdala structure and function have been frequently reported in these disorders [21]–[30].

The amygdala is not a homogenous structure but instead consists of several structurally and functionally heterogeneous nuclei [10], [31]–[34]. For example, one cytoarchitectonic study has divided the amygdala into three subregions: the laterobasal (LB), centromedial (CM) and superficial (SF) regions; this study also established a probabilistic map of these subregions of the amygdala [32]. Based on this model, a task-based functional-MRI study found distinct signal changes in the different amygdala subregions in response to auditory emotional stimuli [33]; furthermore, a resting-state functional-MRI study has demonstrated the distinct rsFC patterns of each of the amygdala subregions [34]. These studies have contributed to our understanding of the functions of these distinct subregions. However, it still remains unclear which amygdala subregion rsFCs are associated with individual HA scores. Thus, we hoped to shed some light on this question by analyzing the correlations between HA scores and the distinct amygdala subregion rsFCs in a large sample of 291 healthy young adults. We also wished to determine any possible gender-based differences in these correlations, which has been frequently reported in HA scores with respect to structural and functional characteristics of the HA-related brain regions [1], [2], [13], [35]–[41].

## Materials and Methods

### Participants

A total of 291 healthy young adults (153 females and 138 males; mean age ± SD = 22.7±2.4 years; range = 18–29 years) were selected from 324 subjects who participated in an imaging/genetic study. The other 33 subjects were excluded from the analyses due to excessive head motion during the fMRI scans. All of the 291 subjects were right-handed [42] native Chinese speakers without any current or past histories of neurologic or psychiatric illnesses; no lesions were observed using conventional brain MRI. The protocol used in this study was approved by the Medical Research Ethics Committee of Tianjin Medical University, and written informed consent was obtained from all participants prior to the experiment.

### Behavioral assessments

HA score was assessed using the Chinese version of the TPQ which has been validated by prior studies [43], [44]. This temperament dimension is characterized by behavioral inhibitions such as pessimistic worrying in anticipation of future problems, passive avoidance behaviors such as fear of uncertainty and shyness of strangers, and rapid fatigability [2]. We also measured and controlled for subclinical depression as assayed using the Beck Depression Inventory (BDI) [45] to exclude the effects of depression on associations between HA scores and amygdala subregion rsFC.

### MRI data acquisition

MR images were acquired using a Signa HDx 3.0 Tesla MR scanner (General Electric, Milwaukee, WI, USA). Tight but comfortable foam padding was used to minimize head motion, and ear plugs were used to reduce scanner noise. Resting-state fMRI data were obtained using Single-Shot Echo-Planar Imaging (SS-EPI) with the following parameters: repetition time (TR)/echo time (TE) = 2000/30 ms; field of view (FOV) = 240 mm×240 mm; matrix = 64×64; flip angle (FA) = 90°; slice thickness = 4 mm, no gap; 40 transversal slices; and 180 volumes. During the fMRI scans, all subjects were instructed to keep their eyes closed, to relax and to move as little as possible, to think of nothing in particular and not to fall asleep. Sagittal 3D T1-weighted images were acquired by a brain volume (BRAVO) sequence (TR/TE = 8.1/3.1 ms, inversion time = 450 ms, FA = 13°, FOV = 256 mm×256 mm, matrix = 256×256, slice thickness = 1 mm, no gap, and 176 sagittal slices).

### MRI data preprocessing

Functional MRI data were analyzed using the statistical parametric-mapping software package SPM8 (http://www.fil.ion.ucl.ac.uk/spm) and the Resting-state fMRI Data Analysis Toolkit REST (v1.6 by Song et al., http://www.restfmri.net) implemented in Matlab R2009 (The Math Works Inc., http://www.mathworks.com). The first 10 volumes of data from each subject were discarded for signal equilibrium and to correct for adaptation of the participants to scanning noise. The remaining 170 volumes were first corrected for any time delays between the acquisition of different slices. Next, head-motion parameters were estimated, and each volume was realigned to the mean map of all the volumes to correct for geometric displacements. Thirty-three of the 324 potential subjects were excluded from further analysis because they had a maximum displacement in any of the orthogonal directions (x, y, z) of more than 2 mm, or a maximum rotation (x, y, z) of more than 2.0°. Then, the data were spatially normalized to the standard EPI template and resampled at 2×2×2 mm voxels. The normalized data were smoothed with a 6 mm full width at half maximum (FWHM) algorithm. After that, linear drift was removed and a temporal filter (0.01–0.08 Hz) was performed to reduce the effect of low-frequency drift and high-frequency noise. Finally, a multiple-regression method was performed to remove possible sources of artifacts, including six estimated motion parameters as well as the average blood oxygen level-dependent (BOLD) signals in the whole brain, ventricular and white matter regions.

### Seed definition

**Figure 1:**
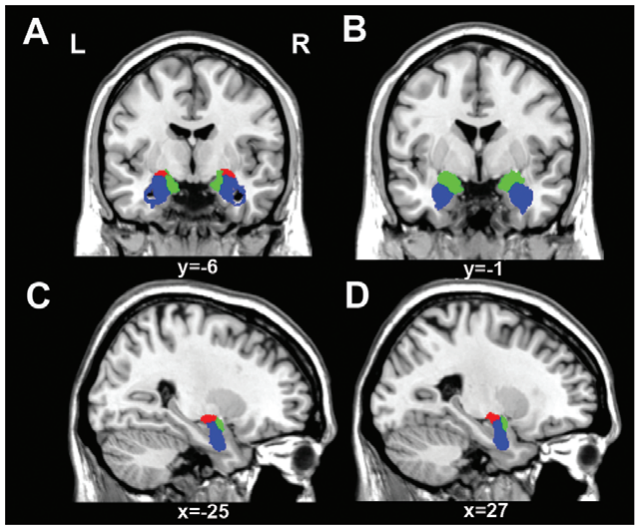
The location of each amygdala subregion (blue = LB; red = CM; and green = SF). A and B show the amygdala subregions of coronal slices y = −6 and y = −1 in Montreal Neurological Institute (MNI) standard space, respectively. C and D show the amygdala subregions of sagittal slices at x = −25 and x = 27 in MNI standard space, respectively. Abbreviations: CM, centromedial subregion; L, left; LB, laterobasal subregion; R, right; and SF, superficial subregion.

The amygdala subregions were extracted using Anatomy v1.7 [46], [47], which provides a maximum probabilistic map (MPM) of each amygdala subregion. The MPM is a summary map of the different regions of the amygdala that aims to attribute each voxel to the most likely subregion with no less than 40% likelihood of cytoarchitectonic probability [46]. The MPM has been confirmed as the best method to defining the amygdala subregions [47] and has been used in several previous studies [28], [48]. Consequently, three non-overlapping MPMs of the amygdala subregions were defined: (1) the LB includes the lateral, basolateral, basomedial, and paralaminar nuclei of the amygdala; (2) the CM consists of the central and medial nuclei; and (3) the SF includes the anterior amygdaloid area, the amygdalopyriform transition area, the amygdaloid-hippocampal area, and the ventral and posterior cortical nuclei (Figure 1).

### Analysis of rsFC

For each participant, the correlation coefficients between the mean time series of each seed and each voxel of the whole brain were computed and converted to z-values using Fisher's r-to-z transformation to improve the normality. The individual z-scaled rsFC maps were entered into a random-effect one-sample *t*-test in a voxel-wise manner to determine the brain regions that showed significantly positive or negative correlations to the seed regions. The significant rsFC maps were corrected for multiple comparisons using the Family Wise Error (FWE) method (*p*<0.05). To exclude insignificant correlations between HA scores and each amygdala subregion rsFC, we restricted the correlation analyses to a mask, which only included brain areas showing significant rsFC with each amygdala subregion.

The relationships between HA scores and amygdala subregion rsFCs were analyzed using a multiple regression model within the significant mask of each amygdala subregion. The age and BDI of the subjects were taken as covariates of no interest to eliminate their potential influences on the results. A correction for multiple comparisons was performed using a Monte Carlo simulation. A threshold of *p*<0.01 for each voxel was used with a cluster size >106 voxels (AlphaSim corrected: 5000 simulations; FWHM = 6 mm; cluster connection radius = 3.3 mm, with gray matter mask; http://afni.nimh.nih.gov/). Finally, the mean z-scaled rsFC values of each significant cluster was extracted and a partial correlation analysis controlling for age and BDI was performed to test for a relationships between HA scores and the z-scaled rsFC of each amygdala subregion in male and female subjects (Bonferroni corrected *p*<0.05).

## Results

### Demographic and behavioral data

The demographic and behavioral data of the subjects are shown in Table 1. A total of 291 young adults (153 females and 138 males) were included in the present study. There were significant differences (*p*<0.05, uncorrected) in age, HA scores and BDI scores between the male and female subjects.

**Table 1:** Demographic and behavioral data of the subjects.

Items	Total subjects	Males	Females	Gender differences
No. of subjects	291	138	153	
Age (years)	22.7±2.4 (18–29)	22.1±2.5 (18–29)	23.2±2.2 (18–29)	*p*<0.05
HA score	14.7±6.5 (1–32)	13.6±6.3 (1–31)	15.8±6.4 (1–32)	*p*<0.05
BDI score	7.9±7.0 (0–52)	9.2±8.0 (0–52)	6.7±5.8 (0–30)	*p*<0.05

Abbreviations: BDI, Beck depression inventory; and HA, harm avoidance.

Data are expressed as the mean ± the standard deviation.

### The patterns of rsFCs of the amygdala subregions

**Figure 2:**
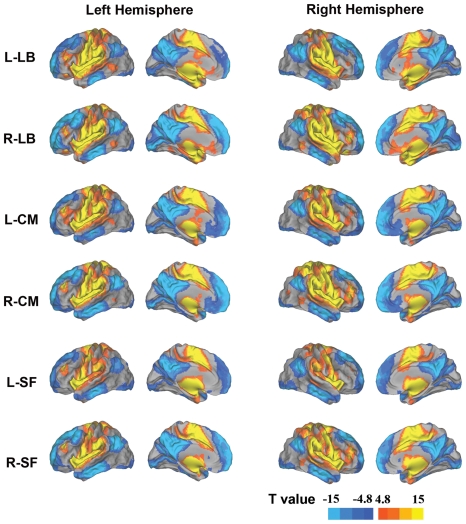
The patterns of rsFC of each amygdala subregion. Warm colors denote positive rsFC values while cold colors denote negative rsFC values. Abbreviations: CM, centromedial subregion; rsFC, resting-state functional connectivity; L, left; LB, laterobasal subregion; R, right; and SF, superficial subregion.

The rsFC patterns of the amygdala subregions are shown in Figure 2. The LB subregion showed positive rsFC bilaterally with the precentral and postcentral gyri, the superior temporal gyri, the insula, and the right supplementary motor area. On the other hand, the LB subregion showed negative rsFC bilaterally with the precuneus lobes, the superior medial frontal gyri, the middle and inferior temporal gyri, the cuneus lobes, the calcarine cortices, and the right angular gyrus. Additionally, the left LB subregion showed negative rsFC with the bilateral superior and middle frontal gyri.

The CM subregion showed positive rsFC bilaterally with the precentral and postcentral gyri, the superior temporal gyri, the insula and supplementary motor areas, and the right supramarginal gyrus. Moreover, the right CM subregion showed positive rsFC with the right middle frontal gyrus and the mid-cingulate cortex. On the other hand, the CM subregion showed negative rsFC bilaterally with the superior and middle frontal gyri, the superior medial frontal gyri, the precuneus lobes, the middle and inferior temporal gyri, and the right angular gyrus.

The SF subregion showed positive rsFC bilaterally with the precentral and postcentral gyri, the superior temporal gyri, the insula, the supplementary motor areas, the supramarginal gyri, the putamens and the mid-cingulate cortices. Furthermore, the right SF subregion showed positive rsFC with the right middle frontal gyrus. On the other hand, the SF subregion showed negative rsFC bilaterally with the superior and middle frontal gyri, the superior medial frontal gyri, the middle and inferior temporal gyri, the right inferior temporal and angular gyri. Additionally, the right SF subregion showed negative rsFC with the right inferior tempral gyrus and the left calcarine cortex.

### Correlations between HA score and rsFCs of the LB

**Figure 3:**
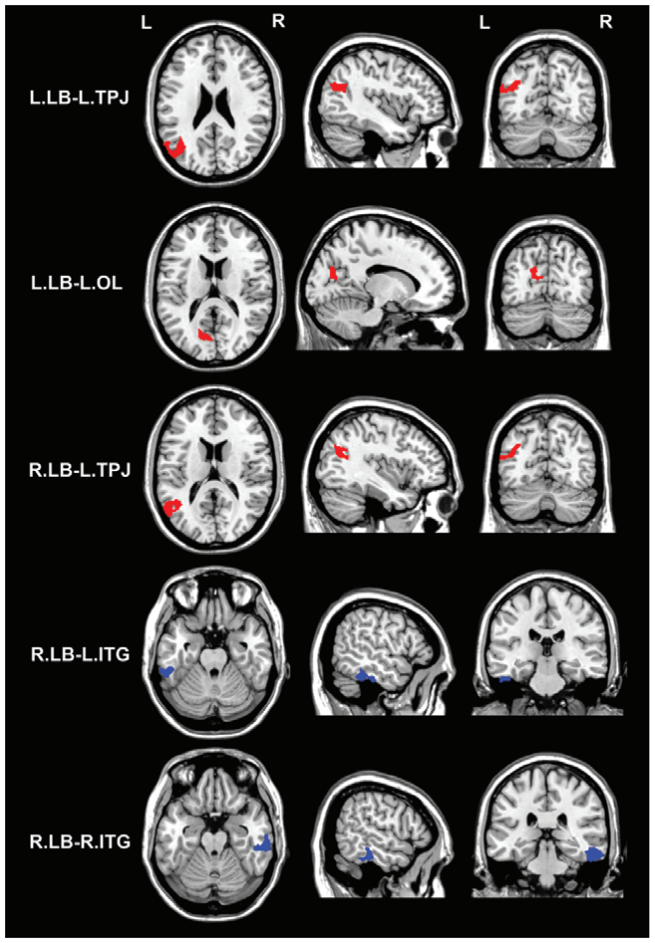
Brain areas in which negative rsFC with the LB is correlated with HA score. Red colors denote positive correlations while blue colors denote negative correlations. Abbreviations: rsFC, resting-state functional connectivity; ITG, inferior temporal gyrus; L, left; LB, laterobasal subregion; OL, occipital lobe; R, right; and TPJ, temporoparietal junction.

The brain areas in which rsFC with the LB was correlated with HA score are shown in Table 2 and Figure 3. None of the positive LB rsFC values were correlated with HA score; however, several of the negative LB rsFC values were correlated with HA score. Specifically, HA scores were positively correlated with rsFCs between the bilateral LB subregions and the left temporoparietal junction (TPJ) (BA39), as well as between the left LB and the left occipital lobe (BA18). However, HA scores were negatively correlated with rsFC between the right LB and the bilateral inferior temporal gyri (ITG) (BA20).

**Table 2:** Brain areas in which rsFC with the LB is correlated with HA score.

rsFC		BA	Cluster size	x	y	z	z values
Patterns	Name						
P	None						
N	L.LB and L.TPJ	39	555	−36	−56	26	4.11
N	L.LB and L. OL	18	206	−14	−74	14	3.41
N	R.LB and L. TPJ	39	479	−56	−54	16	3.96
N	R.LB and L. ITG	20	258	−54	−40	−28	−4.55
N	R.LB and R. ITG	20	373	52	−34	−26	−4.11

Abbreviations: BA, Brodmann Area; rsFC, resting-state functional connectivity; ITG, inferior temporal gyrus; L, left; LB, laterobasal subregion; N, negative; OL, occipital lobe; P, positive; R, right; and TPJ, temporoparietal junction.

### Correlations between HA score and rsFCs of the CM

**Figure 4:**
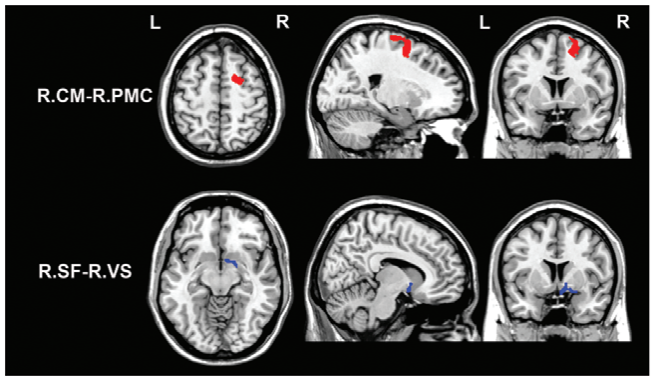
Brain areas in which positive rsFC with the amygdala subregion is correlated with HA score. Among the positive rsFCs with the amygdala subregion, only two were correlated with HA scores. Specifically, HA scores were positively correlated (red colors) with rsFC between the right CM and the right PMC, but were negatively correlated (blue colors) with rsFC between the right SF and the right VS. Abbreviations: CM, centromedial subregion; rsFC, resting-state functional connectivity; L, left; LB, laterobasal subregion; PMC, premotor cortex; R, right; SF, superficial subregion; and VS, ventral striatum.

**Figure 5:**
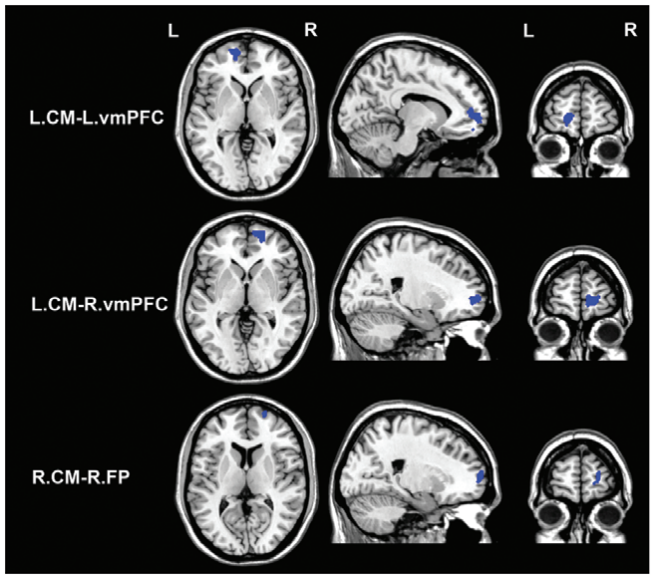
Brain areas in which negative rsFC with the CM is correlated with HA score. Red colors denote positive correlations while blue colors denote negative correlations. Abbreviations: CM, centromedial subregion; rsFC, resting-state functional connectivity; FP, frontal pole; L, left; R, right; and vmPFC, ventromedial prefrontal cortex.

Brain areas in which rsFC with the CM was correlated with HA score are shown in Table 3, Figure 4 and Figure 5. In the areas with positive CM rsFCs, the HA scores were positively correlated with rsFC between the right CM and the right premotor cortex (BA6). In the areas with negative CM rsFCs, the HA scores were negatively correlated with rsFCs between the left CM and the bilateral ventromedial prefrontal cortices (vmPFC) (BA10) as well as between the right CM and the right frontal pole (BA10). However, none of the CM rsFC values were positively correlated with HA score.

**Table 3:** Brain areas in which rsFC with the CM is correlated with HA score.

rsFC		BA	Cluster size	x	y	z	z values
Pattern	Name						
P	R.CM and R. PMC	6	417	24	6	56	3.96
N	L.CM and L.vmPFC	10	182	−14	60	0	−3.88
N	L.CM and R.vmPFC	10	259	16	60	0	−4.27
N	R.CM and R.FP	10	108	22	64	8	−3.38

Abbreviations: BA, Brodmann Area; CM, centromedial subregion; rsFC, resting-state functional connectivity; FP, frontal pole; L, left; N, negative; P, positive; PMC, premotor cortex; R, right; and vmPFC, ventromedial prefrontal cortex.

### Correlations between HA score and rsFCs of the SF

**Figure 6:**
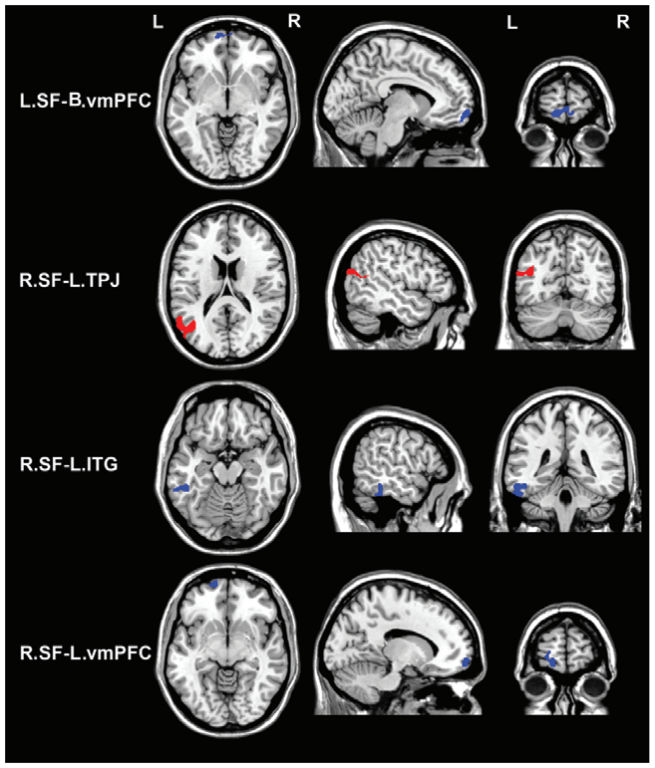
Brain areas in which negative rsFC with the SF is correlated with HA score. Red colors denote positive correlations while blue colors denote negative correlations. Abbreviations: B, bilateral; rsFC, resting-state functional connectivity; ITG, inferior temporal gyrus; L, left; R, right; SF, superficial subregion; TPJ, temporoparietal junction; and vmPFC, ventromedial prefrontal cortex.

Brain areas in which rsFC with the SF was correlated with HA score are shown in Table 4, Figure 4 and Figure 6. In the areas with positive SF rsFC, the HA scores were negatively correlated with rsFC between the right SF and the right ventral striatum. In the areas with negative SF rsFC, the HA scores were negatively correlated with rsFC between the left SF and the bilateral vmPFC as well as between the right SF and the left ITG and the left vmPFC. In contrast, the HA scores were positively correlated with rsFC between the right SF and the left TPJ.

**Table 4:** Brain areas in which rsFC with the SF is correlated with HA score.

rsFC		BA	Cluster size	x	y	z	z values
Pattern	Name						
P	R.SF and R.VS	25	118	22	6	−12	−3.52
N	L.SF and B. vmPFC	10	218	−10	66	−8	−3.79
N	R.SF and L.TPJ	39	428	−42	−66	20	3.61
N	R.SF and L.ITG	20	173	−48	−40	−16	−3.37
N	R.SF and L.vmPFC	10	109	−14	66	−8	−3.82

Abbreviations: BA, Brodmann Area; B, bilateral; rsFC, resting-state functional connectivity; ITG, inferior temporal gyrus; L, left; R, right; SF, superficial subregion; TPJ, temporoparietal junction; vmPFC, ventromedial prefrontal cortex; and VS, ventral striatum.

### Gender-based differences in correlations between HA score and amygdala subregion rsFCs

In the present study, we investigated gender-based differences in the correlations between HA score and amygdala subregion rsFCs as a number of previous studies have reported gender-based differences in HA and HA-related neural substrates [1], [2], [35], [36]. Therefore, we performed a partial-correlation analysis (controlling for age and BDI scores) between HA score and the rsFCs of the amygdala subregions in male and female subjects (Table 5). As shown in Table 5, we found the following: (1) correlations between HA score and LB rsFCs were stronger in females; (2) correlations between HA score and CM rsFCs were stronger in males; and (3) no sex-based differences in correlations between HA score and SF rsFCs. A Bonferroni-corrected threshold of p<0.05 corresponded to an uncorrected threshold of p<0.0036 (0.05/14). In male subjects, individual HA score could be predicted by rsFC values between the right LB and the right ITG, between the right CM and the right frontal pole, and between the right CM and the right premotor cortex. In contrast, in female subjects, individual HA scores could be predicted by rsFC values between the left LB and the left TPJ and occipital lobe, between the right LB and the left ITG, between the left CM and the left vmPFC, and between the right SF and the right ventral striatum.

**Table 5:** Gender-based differences in correlations between HA score and amygdala subregion rsFC.

rsFC	Male subjects (n = 138)		Female subjects (n = 153)	
	PCC	*p*	PCC	*p*
**Positive rsFCs**				
R.CM and R. PMC	**0.378**	<0.001*	0.085	0.302
R.SF and R.VS	−0.233	0.006	**−0.245**	0.002*
**Negative rsFCs**				
L.LB and L.TPJ	0.143	0.097	**0.269**	0.001*
L.LB and L. OL	0.176	0.041	**0.267**	0.001*
R.LB and L. TPJ	0.108	0.212	0.230	0.005
R.LB and L. ITG	−0.176	0.041	**−0.244**	0.003*
R.LB and R. ITG	**−0.255**	0.003*	−0.203	0.012
L.CM and L.vmPFC	−0.193	0.024	**−0.240**	0.003*
L.CM and R.vmPFC	−0.224	0.009	−0.211	0.009
R.CM and R.FP	**−0.362**	<0.001*	−0.012	0.886
L.SF and B. vmPFC	−0.244	0.004	−0.162	0.047
R.SF and L.TPJ	0.175	0.041	0.197	0.015
R.SF and L.ITG	−0.168	0.049	−0.208	0.010
R.SF and L.vmPFC	−0.209	0.015	−0.230	0.005

Abbreviations: B, bilateral; CM, centromedial subregion; rsFC, resting-state functional connectivity; FP, frontal pole; ITG, inferior temporal gyrus; L, left; LB, laterobasal subregion; OL, occipital lobe; PMC, premotor cortex; PCC, Partial Correlation Coefficient; R, right; SF, superficial subregion; TPJ, temporoparietal junction; vmPFC, ventromedial prefrontal cortex; and VS, ventral striatum. Asterisks (*) denote a significant correlation between HA score and amygdala subregion rsFC after Bonferroni correction for multiple comparisons.

## Discussion

In the present study, we investigated the contributions of amygdala subregion rsFCs to individual HA scores in a large sample of 291 healthy young adults. We found that individual HA scores could be predicted by the rsFCs between the LB and temporal and occipital areas, between the CM and the frontal areas, and between the SF and the frontal and temporal areas, which confirms the association between HA scores and several specific amygdala subregion rsFCs. We also found gender-based differences in these correlations, which is also consistent with previous reports.

### Possible functional implications of correlations between HA score and rsFC

One should be cautious when interpreting negative rsFC values derived from resting-state fMRI studies, as it remains an unsettled debate whether negative rsFC is an artifact of global signal regression [49], [50] or if it in fact reflects dynamic anti-correlated functional networks [51]. Functional connectivity has the ability to determine how much of the activity or activation variance in one region is explained by another region, while the polarity of the connectivity may be relevant but of secondary importance [20]. A positive polarity is suggestive of a facilitatory influence while a negative polarity may indicate a regulatory influence [20]. The positive correlation between HA score and positive rsFC suggests that individuals with high HA scores have increased facilitatory influence between two brain regions, and vice versa for negative correlations. In contrast, a negative correlation between HA score and negative rsFC suggests that individuals with high HA scores have increased regulatory influence between the two brain regions, and vice versa for positive correlations. The following discussions will be based on these hypotheses.

### Correlations between HA score and LB rsFCs

The LB is the largest of the three amygdala subregions and is the main nucleus that receives sensory inputs from the visual, auditory, somatosensory, and olfactory systems [10], [31], [52]. The LB is thought to play a pivotal role in assigning emotional value to sensory stimuli [31] and promoting associative-learning processes such as fear conditioning through afferents from the cortical and subcortical regions [12], [53]. Activation of the amygdala in response to auditory emotional stimuli was mainly located in the LB subregion [33], which also supports the role of this subregion in receiving inputs from sensory systems.

We found that HA scores were positively correlated with a negative rsFC between the left LB and the left visual cortex, which suggests that individuals with high HA scores have decreased regulatory influence between the brain areas related to emotional evaluation (LB) and perception (visual cortex). Associations between HA scores and the visual cortex have also been reported in a previous resting-state perfusion study [54] and a VBM study [55]. The TPJ can integrate multisensory information to encode the subjective feelings of the self [56] and is vital for interpreting the thoughts, feelings, and mental state of others [57], [58]. The positive correlation between HA score and a negative rsFC between the LB and TPJ also suggests a reduced regulatory influence between these two brain areas, which are key structures in the processing of social cognition [11], [59], [60]. Although the ITG has been reported to be part of the ventral visual pathway and is involved in the processing of language and memory [61]–[64], the relationship of the ITG with emotion has also been appreciated both in healthy subjects [65] as well as in patients with depression [66], [67] or anxiety disorders [68], [69]. These findings suggest that both the LB and ITG are implicated in emotional processing, and indeed their functional organizations have been previously correlated with HA scores [70]. The negative correlation between HA score and negative rsFC between the LB and ITG regions suggests that individuals with high HA scores have increased regulatory influence between these two regions, which could cause a predisposition towards mood disorders.

### Correlations between HA score and CM rsFCs

The CM subregion receives inputs from other subregions of the amygdala, the thalamus, the brain stem and several cortical areas, and it then sends efferents to various subcortical structures in order to control the affective-related behavioral responses [53], [71]. The CM is believed to be an important “output” amygdala subregion and an interface of the amygdala with the motor system, which is closely related to the expression of innate emotional responses and associated physiological responses [53], [71]. Since the premotor cortex is associated with planning motor actions [72], our finding that a positive correlation exists between HA score and a positive rsFC between the CM and the premotor cortex suggests that CM activity in individuals with high HA scores has an increased influence on the motor cortex, which might explain the increased behavioral responses to aversive stimuli in these subjects.

A large number of studies have demonstrated a prominent role for the vmPFC in emotional regulation and social-cognitive functions [73], [74]. The CM subregion of the amygdala can receive modulatory signals from the vmPFC through the anterior cingulate cortex [10], [20], [73], [75], which can then affect the emotional responses and associated physiological responses mediated by this subregion of the amygdala. The association between HA score and the vmPFC has also been reported by two positron-emission tomography studies [41], [76]. We found that HA scores were negatively correlated with a negative rsFC between the CM subregion and the vmPFC, which is consistent with a previous study reporting that HA scores can be predicted by the task-state functional coupling between the amygdala and the vmPFC [20].

### Correlations between HA score and SF rsFCs

The SF consists of the anterior amygdala area, the amygdalopyriform transition area, the amygdaloid-hippocampal area and the ventral and posterior cortical nuclei, and it is one of the less-studied amygdala subregions. In lower nonprimate animals, the SF has been functionally linked with intraspecies communication via olfactory stimuli [77]. However, in human brain, by combining fMRI techniques with the cytoarchitectonic-probabilistic maps of the human amygdala subregions, several studies have shown that the SF subregion is involved in social-emotional information processing, in particular with facial expressions [48], [78]. Similarly to that seen with the CM, the functional organization of the SF may partially account for our finding that a correlation exists between HA scores and the rsFC between the SF and the vmPFC. Similarly to that seen with the LB, we also found correlations between the SF and the rsFCs of the SF with the TPJ and ITG, suggesting these two subregions (i.e., the SF and LB) may share some common functional organizations; this is consistent with the comparative architectonic-based classification created by separating the cortical amygdaloid nucleus (the SF component) from the CM group and assigning it to the LB group and with the observation of similar evolution trajectories for these two subregions in an ascending primate scale [79]. In addition, we found that HA scores were correlated with rsFC between the right SF subregion and the right ventral striatum, which are anatomically connected areas. Indeed, the ventral striatum may be implemented in tasks involving emotion and motivation through connections with the amygdala [80]. The negative correlation between HA score and rsFC between these two areas suggests that decreases in this rsFC may predispose individuals for high HA scores. The association between HA score and the ventral striatum has also been observed in a previous fMRI study [81].

### Sex-based differences in correlations between HA score and amygdala subregion rsFC

Sex-based differences have been found in HA scores [1], [2], [35], [36], amygdala activation during emotional tasks [82]–[87], glucose metabolism in the amygdala during rest [88], amygdala rsFCs [89], correlations between HA scores and amygdala volume [13], metabolism during rest [41], and task-state functional coupling between the amygdala and prefrontal cortex [20]. In the present study, we found that females showed stronger correlations between HA score and LB rsFC, while males had stronger correlations between HA score and CM rsFC. These findings suggest that HA score is associated with rsFCs of the amygdala subregions implicated in emotional input and evaluation in females but that it is associated with rsFCs of the amygdala subregions related to emotional output and regulation in males. Although the mechanisms underlying these sex-based differences in the associations between HA scores and amygdala rsFCs remain unclear, they might be partly explained by differences in genetic variants [20], hormone levels [90], [91], or the different responsive modes to emotional stimuli between males and females [83], [86], [92].

### Limitations

Several limitations of the present study should be considered when interpreting our results. Firstly, the resolution of a 64×64 matrix is quite low for small structures, such as the amygdala subregions. The selection of the fMRI parameters such as the imaging matrix is a trade-off process. On the basis of the imaging technique we are available; a higher matrix such as 128×128 can inevitably improve the image resolution and show more details of structures of interest, such as the amygdala subregions. However, in the single-shot EPI sequence, the increase of the matrix will lead to severe image distortion, especially in structures (such as the amygdala) near the skull base. Therefore, we selected an imaging resolution of a 64×64 matrix to study the amygdala subregions as has been adopted in most of the previous studies on the amygdala subregions [28], [33], [34], [48]. Secondly, despite partial correlation controlling for age and BDI can reduce the influences of these two variables on our correlation analyses between amygdala rsFCs and HA scores, we cannot absolutely exclude the effects of the significant group differences in age, BDI, and HA on our partial correlation analyses. Finally, the standard smoothing procedures may result in the contamination of one amygdala subregion by the neighboring subregions and other nearby brain structures. To clarify the issue, we calculated the rsFCs using unsmoothed fMRI data (see Supporting Information S1) and found that most of our current findings were replicated in the new analysis with unsmoothed fMRI data (see Supporting Information S2, Figures S1, S2, S3, and S4). This analysis confirms the reliability of our results using smoothed fMRI data.

### Conclusion

In summary, we recruited a large sample of healthy young adults to investigate the differential contributions of amygdala subregion rsFCs to individual HA scores. We found that individual HA scores could be predicted by the rsFC between each of the amygdala subregions to a number of specific connected brain regions. These findings not only extend the number of confirmed associations between HA score and structural and functional properties of amygdala rsFC, but they also extend these associations to a subregion-specific level. We also found sex-based differences in these correlations, which extends our knowledge of sex-based differences in the correlations between HA score and amygdala properties to a subregion-specific level.

## Supporting Information

Figure S1 Brain areas in which negative rsFC with the LB is correlated with HA score. Red colors denote positive correlations while blue colors denote negative correlations. Abbreviations: rsFC, resting-state functional connectivity; ITG, inferior temporal gyrus; L, left; LB, laterobasal subregion; OL, occipital lobe; R, right; and TPJ, temporoparietal junction.
(DOC)

Figure S2 Brain areas in which positive rsFC with the amygdala subregion is correlated with HA score. Among the positive rsFCs with the amygdala subregion, only two were correlated with HA scores. Specifically, HA scores were positively correlated (red colors) with rsFC between the right CM and the right PMC, but were negatively correlated (blue colors) with rsFC between the right SF and the right VS. Abbreviations: CM, centromedial subregion; rsFC, resting-state functional connectivity; L, left; LB, laterobasal subregion; PMC, premotor cortex; R, right; SF, superficial subregion; and VS, ventral striatum.
(DOC)

Figure S3 Brain areas in which negative rsFC with the CM is correlated with HA score. Blue colors denote negative correlations. Abbreviations: CM, centromedial subregion; rsFC, resting-state functional connectivity; L, left; R, right; and vmPFC, ventromedial prefrontal cortex.
(DOC)

Figure S4 Brain areas in which negative rsFC with the SF is correlated with HA score. Blue colors denote negative correlations. Abbreviations: B, bilateral; rsFC, resting-state functional connectivity; ITG, inferior temporal gyrus; L, left; R, right; SF, superficial subregion; and vmPFC, ventromedial prefrontal cortex.
(DOC)

Supporting Information S1 Correlation analyses between HA score and rsFCs of the amygdala subregions using unsmoothed fMRI data: Background and methods.
(DOC)

Supporting Information S2 Correlation analyses between HA score and rsFCs of the amygdala subregions using unsmoothed fMRI data: Results.
(DOC)
