# Supplementary material for: Sex-Dependent Correlations between the Personality Dimension of Harm Avoidance and the Resting-State Functional Connectivity of Amygdala Subregions
Source: PLoS One. 2012 Apr 27;7(4):e35925. doi: 10.1371/journal.pone.0035925 (PMC3338761; doi:10.1371/journal.pone.0035925)
Supplement: Figure S1 — Brain areas in which negative rsFC with the LB is correlated with HA score. Red colors denote positive correlations while blue colors denote negative correlations. Abbreviations: rsFC, resting-state functional connectivity; ITG, inferior temporal gyrus; L, left; LB, laterobasal subregion; OL, occipital lobe; R, right; and TPJ, temporoparietal junction. (DOC) [file pone.0035925.s001.doc]

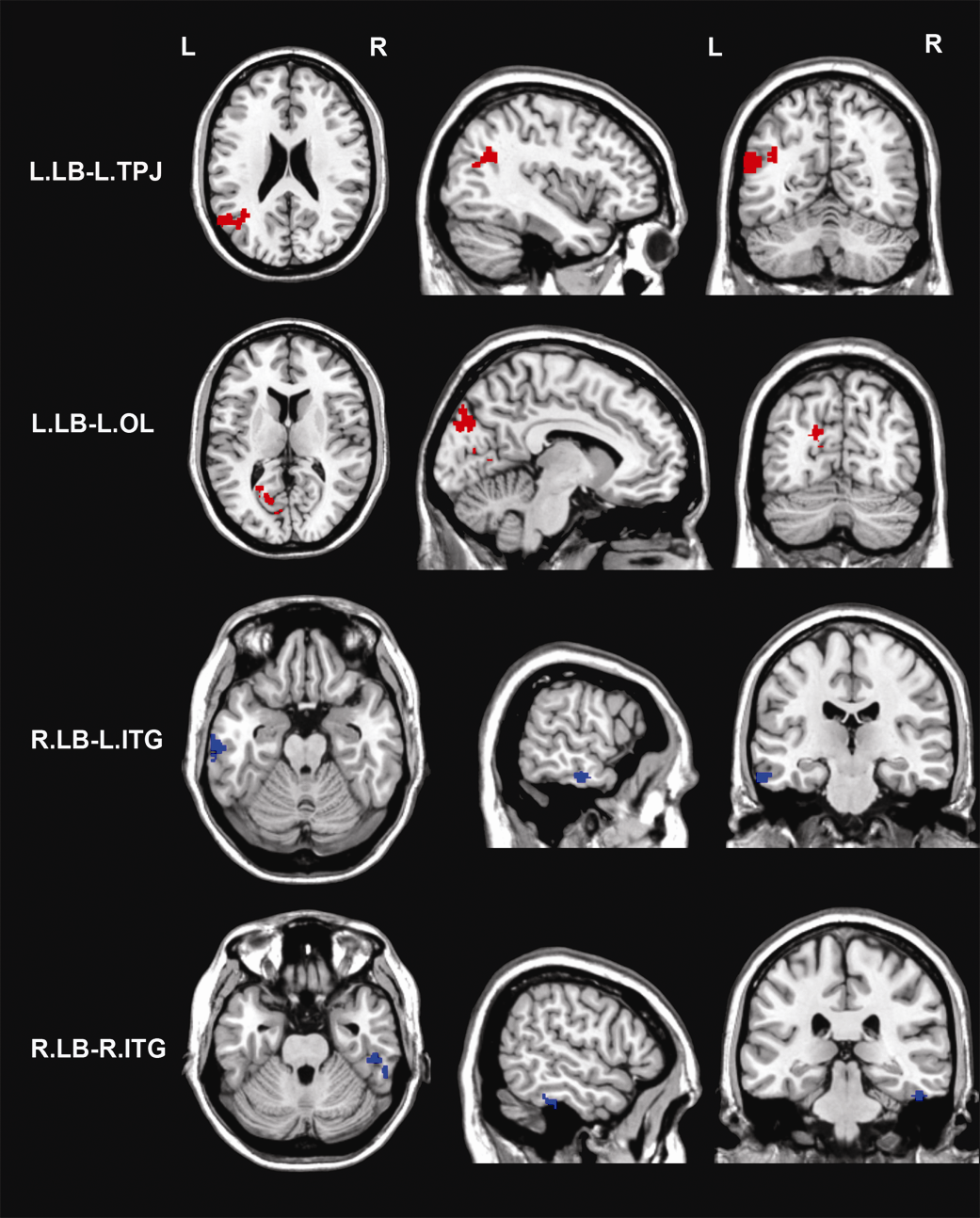


**Fig S1.** Brain areas in which negative rsFC with the LB is correlated with HA score. Red colors denote positive correlations while blue colors denote negative correlations. Abbreviations: rsFC, resting-state functional connectivity; ITG, inferior temporal gyrus; L, left; LB, laterobasal subregion; OL, occipital lobe; R, right; and TPJ, temporoparietal junction.
