# Supplementary material for: Sex-Dependent Correlations between the Personality Dimension of Harm Avoidance and the Resting-State Functional Connectivity of Amygdala Subregions
Source: PLoS One. 2012 Apr 27;7(4):e35925. doi: 10.1371/journal.pone.0035925 (PMC3338761; doi:10.1371/journal.pone.0035925)
Supplement: Figure S2 — Brain areas in which positive rsFC with the amygdala subregion is correlated with HA score. Among the positive rsFCs with the amygdala subregion, only two were correlated with HA scores. Specifically, HA scores were positively correlated (red colors) with rsFC between the right CM and the right PMC, but were negatively correlated (blue colors) with rsFC between the right SF and the right VS. Abbreviations: CM, centromedial subregion; rsFC, resting-state functional connectivity; L, left; LB, laterobasal subregion; PMC, premotor cortex; R, right; SF, superficial subregion; and VS, ventral striatum. (DOC) [file pone.0035925.s002.doc]

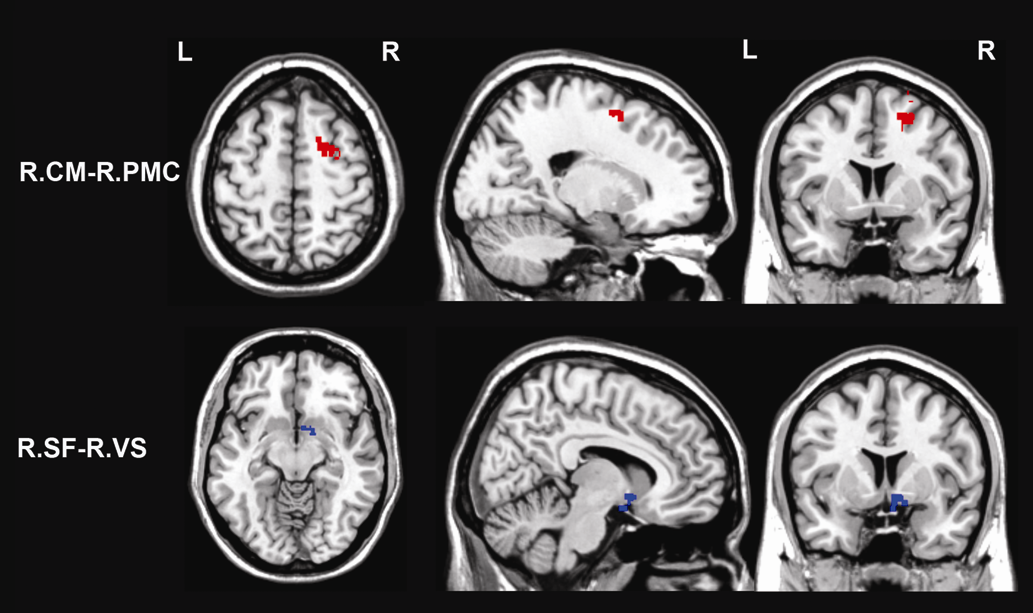


**Figure S2.** Brain areas in which positive rsFC with the amygdala subregion is correlated with HA score. Among the positive rsFCs with the amygdala subregion, only two were correlated with HA scores. Specifically, HA scores were positively correlated (red colors) with rsFC between the right CM and the right PMC, but were negatively correlated (blue colors) with rsFC between the right SF and the right VS. Abbreviations: CM, centromedial subregion; rsFC, resting-state functional connectivity; L, left; LB, laterobasal subregion; PMC, premotor cortex; R, right; SF, superficial subregion; and VS, ventral striatum.

x.
