# Supplementary material for: Sex-Dependent Correlations between the Personality Dimension of Harm Avoidance and the Resting-State Functional Connectivity of Amygdala Subregions
Source: PLoS One. 2012 Apr 27;7(4):e35925. doi: 10.1371/journal.pone.0035925 (PMC3338761; doi:10.1371/journal.pone.0035925)
Supplement: Figure S3 — Brain areas in which negative rsFC with the CM is correlated with HA score. Blue colors denote negative correlations. Abbreviations: CM, centromedial subregion; rsFC, resting-state functional connectivity; L, left; R, right; and vmPFC, ventromedial prefrontal cortex. (DOC) [file pone.0035925.s003.doc]

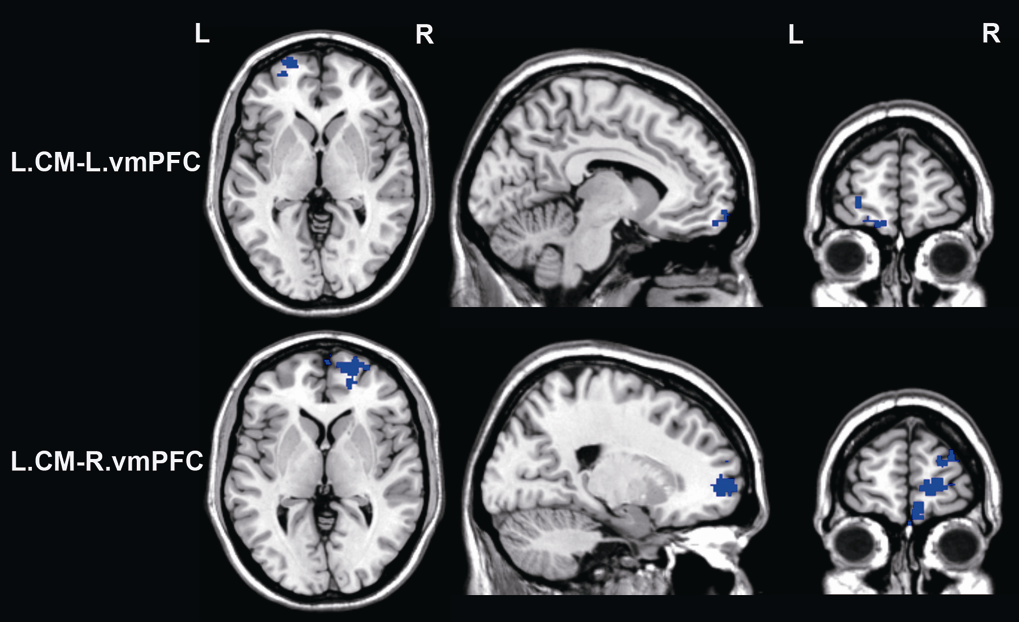


**Figure S3.** Brain areas in which negative rsFC with the CM is correlated with HA score. Blue colors denote negative correlations. Abbreviations: CM, centromedial subregion; rsFC, resting-state functional connectivity; L, left; R, right; and vmPFC, ventromedial prefrontal cortex.
