# Supplementary material for: Sex-Dependent Correlations between the Personality Dimension of Harm Avoidance and the Resting-State Functional Connectivity of Amygdala Subregions
Source: PLoS One. 2012 Apr 27;7(4):e35925. doi: 10.1371/journal.pone.0035925 (PMC3338761; doi:10.1371/journal.pone.0035925)
Supplement: Figure S4 — Brain areas in which negative rsFC with the SF is correlated with HA score. Blue colors denote negative correlations. Abbreviations: B, bilateral; rsFC, resting-state functional connectivity; ITG, inferior temporal gyrus; L, left; R, right; SF, superficial subregion; and vmPFC, ventromedial prefrontal cortex. (DOC) [file pone.0035925.s004.doc]

**
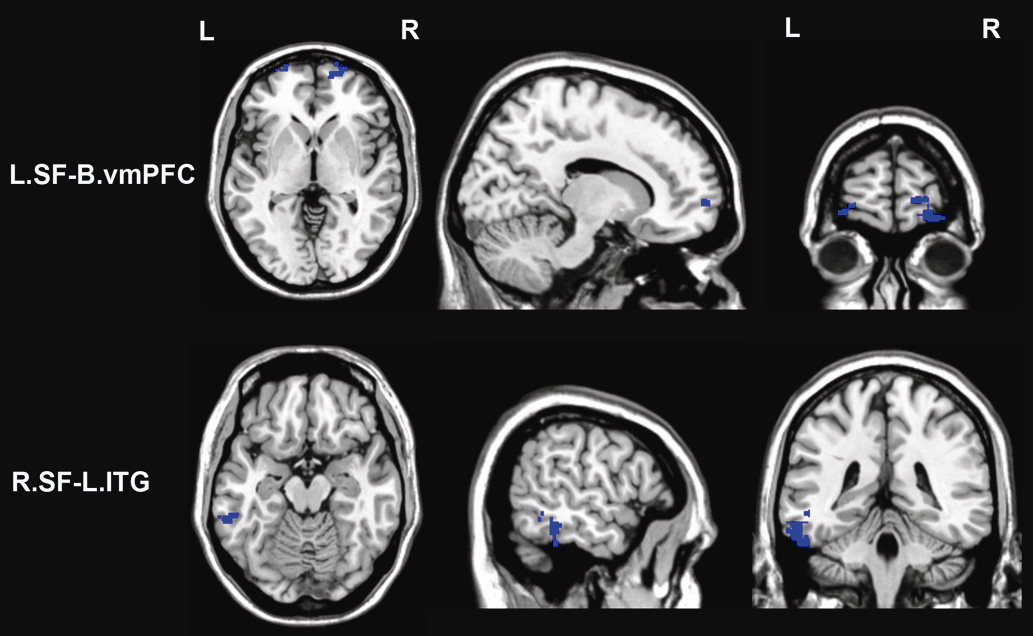
**

**Figure S4.** Brain areas in which negative rsFC with the SF is correlated with HA score. Blue colors denote negative correlations. Abbreviations: B, bilateral; rsFC, resting-state functional connectivity; ITG, inferior temporal gyrus; L, left; R, right; SF, superficial subregion; and vmPFC, ventromedial prefrontal cortex.
