# Supplementary material for: Sex-Dependent Correlations between the Personality Dimension of Harm Avoidance and the Resting-State Functional Connectivity of Amygdala Subregions
Source: PLoS One. 2012 Apr 27;7(4):e35925. doi: 10.1371/journal.pone.0035925 (PMC3338761; doi:10.1371/journal.pone.0035925)
Supplement: Supporting Information S1 — Correlation analyses between HA score and rsFCs of the amygdala subregions using unsmoothed fMRI data: Background and methods. (DOC) [file pone.0035925.s005.doc]

**Supporting Information S1**

**Correlation analyses between HA score and rsFCs of the amygdala subregions using unsmoothed fMRI data**

**Background**

The standard smoothing procedures may result in the contamination of one amygdala subregion by the neighboring subregions and other nearby brain structures. To clarify the issue, we calculated the rsFCs of each amygdala subregions using unsmoothed fMRI data and other preprocessing steps remained the same as we used in the analysis of smoothed fMRI data.

**Methods**

***MRI data preprocessing***

Functional MRI data were analyzed using the statistical parametric-mapping software package SPM8 (<http://www.fil.ion.ucl.ac.uk/spm>) and the Resting-state fMRI Data Analysis Toolkit REST (v1.6 by Song et al., [http://www.restfmri.net](http://www.restfmri.net/)) implemented in Matlab R2009 (The Math Works Inc., [http://www.mathworks.com](http://www.mathworks.com/)). The first 10 volumes of data from each subject were discarded for signal equilibrium and to correct for adaptation of the participants to scanning noise. The remaining 170 volumes were first corrected for any time delays between the acquisition of different slices. Next, head-motion parameters were estimated, and each volume was realigned to the mean map of all the volumes to correct for geometric displacements. Thirty-three of the 324 potential subjects were excluded from further analysis because they had a maximum displacement in any of the orthogonal directions (x, y, z) of more than 2 mm, or a maximum rotation (x, y, z) of more than 2.0°. Then, the data were spatially normalized to the standard EPI template and resampled at 2 × 2 × 2 mm voxels. The normalized data were not smoothed. After that, linear drift was removed and a temporal filter (0.01-0.08 Hz) was performed to reduce the effect of low-frequency drift and high-frequency noise. Finally, a multiple-regression method was performed to remove possible sources of artifacts, including six estimated motion parameters as well as the average blood oxygen level-dependent (BOLD) signals in the whole brain, ventricular and white matter regions.

***Seed definition***

The amygdala subregions were extracted using Anatomy v1.7, which provides a maximum probabilistic map (MPM) of each amygdala subregion. Three non-overlapping MPMs of the amygdala subregions were defined: (1) the LB includes the lateral, basolateral, basomedial, and paralaminar nuclei of the amygdala; (2) the CM consists of the central and medial nuclei; and (3) the SF includes the anterior amygdaloid area, the amygdalopyriform transition area, the amygdaloid-hippocampal area, and the ventral and posterior cortical nuclei.

***Analysis of rsFC***

For each participant, the correlation coefficients between the mean time series of each seed and each voxel of the whole brain were computed and converted to z-values using Fisher's r-to-z transformation to improve the normality. The individual z-scaled rsFC maps were entered into a random-effect one-sample *t*-test in a voxel-wise manner to determine the brain regions that showed significantly positive or negative correlations to the seed regions. The significant rsFC maps were corrected for multiple comparisons using the Family Wise Error (FWE) method (*p* < 0.05). To exclude insignificant correlations between HA scores and each amygdala subregion rsFC, we restricted the correlation analyses to a mask, which only included brain areas showing significant rsFC with each amygdala subregion.

The relationships between HA scores and amygdala subregion rsFCs were analyzed using permutation statistical analysis (a non-parametric statistical method; ‘randomise’ script embedded in FSL <http://www.fmrib.ox.ac.uk/fsl>, 5000 permutation) within the significant mask of each amygdala subregion. The age and BDI of the subjects were taken as covariates of no interest to eliminate their potential influences on the results. The result was corrected for multiple correction using Threshold-Free Cluster Enhancement (TFCE) methods with *p* < 0.01.
