# Supplementary material for: Sex-Dependent Correlations between the Personality Dimension of Harm Avoidance and the Resting-State Functional Connectivity of Amygdala Subregions
Source: PLoS One. 2012 Apr 27;7(4):e35925. doi: 10.1371/journal.pone.0035925 (PMC3338761; doi:10.1371/journal.pone.0035925)
Supplement: Supporting Information S2 — Correlation analyses between HA score and rsFCs of the amygdala subregions using unsmoothed fMRI data: Results. (DOC) [file pone.0035925.s006.doc]

**Supporting Information S1**

**Correlation analyses between HA score and rsFCs of the amygdala subregions using unsmoothed fMRI data**

**Results**

***Correlations between HA score and rsFCs of the LB***

The brain areas in which rsFC with the LB was correlated with HA score are shown in Figure S1. None of the positive LB rsFC values were correlated with HA score; however, several of the negative LB rsFC values were correlated with HA score. Specifically, HA scores were positively correlated with rsFCs between the left LB subregions and the left temporoparietal junction (TPJ) (BA39), as well as between the left LB and the left occipital lobe (BA18). However, HA scores were negatively correlated with rsFC between the right LB and the bilateral inferior temporal gyri (ITG) (BA20).

***Correlations between HA score and rsFCs of the CM***

Brain areas in which rsFC with the CM was correlated with HA score are shown in Figure S2 and Figure S3. In the areas with positive CM rsFCs, the HA scores were positively correlated with rsFC between the right CM and the right premotor cortex (BA6). In the areas with negative CM rsFCs, the HA scores were negatively correlated with rsFCs between the left CM and the bilateral ventromedial prefrontal cortices (vmPFC) (BA10). However, none of the CM rsFC values were positively correlated with HA score.

***Correlations between HA score and rsFCs of the SF***

Brain areas in which rsFC with the SF was correlated with HA score are shown in Figure S2 and Figure S4. In the areas with positive SF rsFC, the HA scores were negatively correlated with rsFC between the right SF and the right ventral striatum. In the areas with negative SF rsFC, the HA scores were negatively correlated with rsFC between the left SF and the bilateral vmPFC as well as between the right SF and the left ITG.
